# Modeling Periodic Impulsive Effects on Online TV Series Diffusion

**DOI:** 10.1371/journal.pone.0163432

**Published:** 2016-09-26

**Authors:** Peihua Fu, Anding Zhu, Qiwen Fang, Xi Wang

**Affiliations:** College of Computer and Information Engineering, Zhejiang Gongshang University, Hangzhou, Zhejiang, P.R. China; Hangzhou Normal University, CHINA

## Abstract

**Background:**

Online broadcasting substantially affects the production, distribution, and profit of TV series. In addition, online word-of-mouth significantly affects the diffusion of TV series. Because on-demand streaming rates are the most important factor that influences the earnings of online video suppliers, streaming statistics and forecasting trends are valuable. In this paper, we investigate the effects of periodic impulsive stimulation and pre-launch promotion on on-demand streaming dynamics. We consider imbalanced audience feverish distribution using an impulsive susceptible-infected-removed(SIR)-like model. In addition, we perform a correlation analysis of online buzz volume based on Baidu Index data.

**Methods:**

We propose a PI-SIR model to evolve audience dynamics and translate them into on-demand streaming fluctuations, which can be observed and comprehended by online video suppliers. Six South Korean TV series datasets are used to test the model. We develop a coarse-to-fine two-step fitting scheme to estimate the model parameters, first by fitting inter-period accumulation and then by fitting inner-period feverish distribution.

**Results:**

We find that audience members display similar viewing habits. That is, they seek new episodes every update day but fade away. This outcome means that impulsive intensity plays a crucial role in on-demand streaming diffusion. In addition, the initial audience size and online buzz are significant factors. On-demand streaming fluctuation is highly correlated with online buzz fluctuation.

**Conclusion:**

To stimulate audience attention and interpersonal diffusion, it is worthwhile to invest in promotion near update days. Strong pre-launch promotion is also a good marketing tool to improve overall performance. It is not advisable for online video providers to promote several popular TV series on the same update day. Inter-period accumulation is a feasible forecasting tool to predict the future trend of the on-demand streaming amount. The buzz in public social communities also represents a highly correlated analysis tool to evaluate the advertising value of TV series.

## Introduction

TV series that are consist of a limited number of episodes are a popular type of entertainment welcomed by all ages. TV series were traditionally broadcast on television until *House of Cards*, a popular American political drama TV series first broadcast in 2013, was released in full through the Netfilex streaming service [1]. In China, several major online video-streaming service suppliers buy the broadcast rights of TV series and sell inserted commercial advertisements, which audiences must view. To optimize their advertisement schedules, it is important for video-on-demand (VOD) suppliers to record on-demand streaming statistics and forecast corresponding trends.

As social network services (SNSs) have become widespread over the past few years, audiences have become accustomed to discussing and sharing the plots of series and posting valence (i.e., a positive or a negative sentiment) in online social communities. With the help of behavioral e-footprints of audiences, VOD suppliers have the opportunities to learn the collective behaviors of human dynamics by modern computational social science approaches [2, 3]. Several studies suggest that the buzz or word-of-mouth volume in social networks can be used to forecast a movie’s box-office performance [4, 5].

According to the Bass model, which is a traditional, concise innovation diffusion model proposed by Bass in 1969 [6] to forecast the first adoption of durable products, there are two types of agent: innovators and imitators. Each type has an effect on the adoption diffusion process. Theoretically, the Bass model consists of a simple non-linear differential equation of the adoption function *F*(*t*) with respect to time *t*: $\frac{\partial F(t)}{\partial t}=\left[p+qF\left(t\right)\right]\left[1-F\left(t\right)\right]$, where *p* is the coefficient of innovation from external influences and *q* is the coefficient of imitation from internal influences. This equation has a counterpart in epidemic-spread theory, whereby *q* corresponds to the spreading rate in the standard susceptible-infected (SI) equation of the infection function *I*(*t*): $\frac{\partial I(t)}{\partial t}=qI\left(t\right)\left[1-I\left(t\right)\right]$ and if we assume susceptible agents will be infected independently and randomly from outside. Thus, the Bass model can be regarded as an *imported SI model*.

A large body of literature has adopted biological SI-family models to study peer-wise cascade diffusion phenomena in the multidisciplinary branches of several fields, including physics, sociology, economics, and information science [7]. In particular, the theoretical result for zero-tended critical thresholds for restraining epidemic outbreaks in scale-free complex networks [8] has inspired researchers to investigate the collective features of human dynamics in social networks, such as rumor diffusion [9–11], information sharing [12, 13], and word-of-mouth contagion [14].

Most of these studies assume that the epidemic-like diffusion occurs in a closed system. That is, during the initial stage, only few infected individuals become disseminative origins who trigger diffusion, and the system takes no external influences into account [15]. However, Myer et al. find that only 71% of Twitter’s tweet volume is attributed to network diffusion. The remaining 29% is due to external events and factors external to the network [16]. Stated simply, the literature can distinguish agents who are randomly infected by outside origins from interpersonally infected ones [17, 18].

In comparison, TV series exert another type of imported influence on diffusion process. TV series update periods are fixed, which can be viewed as a periodic impulsive stimulation of the entire audiences. These circumstances resemble the impulsive vaccination of the susceptible-infected-removed(SIR)-like epidemic model in biology [19–21]. However, impulsive vaccination suppresses epidemic diffusion, whereas TV series updates stimulate diffusion.

On the other hand, audiences have no chance to view the episodes they missed in the conventional television broadcasting mode, while they can review any times they want in the online steaming mode. There are so many video seeds in the online platform leading to choice overload for audiences. Li et al. introduce the concept of view scope to model the user information-processing capability under information overload in the Facebook- [22, 23] and Twitter-like social networks [24]. Su et al. investigate the incomplete reading behavior of microblog users encountering massive messages by improving the traditional epidemic model with a reading rate [25]. Wang et al. model the nonredundant information transmission behavior in social networks [26]. However, our study finds that the periodic impulsive stimulation would influence the online streaming behavior of TV series audience significantly because of the lock-in effect. Therefore, we develop a *periodic impulsive—susceptible-infected-recovered* (PI-SIR) model to analyze the periodic impulsive effects on online TV series diffusion and test this model using three popular South Korean TV series.

## Analysis

### The basic model

Referring to the basic SIR epidemic diffusion deterministic model proposed by McKendrick and Kermack [27], we denote *S*(*t*) as the number of agents who have not yet been attracted by the TV series at time *t*, *I*(*t*) as the number of agents who have adopted the TV series and might share information with or spread valence to their friends in social networks or with physical contacts and *R*(*t*) as the number of agents who have lost interest in the TV series and give up watching.

Zhou et al. demonstrate that the social network has a heterogeneous topological structure with scale-free distribution of node degrees [28], which means that the minority of the population has larger connectivity and influence than the remaining majority. Wang et al. find the heterogeneous network structure has a significant influence on the epidemic threshold and final size [29]. However, in modern society, people live in a complicated communication network in which they can keep in touch with one another online or offline.

Since it is difficult to reshape the influence relationship among audience, to simplify, we assume that mixed communication networks have a homogeneous network structure for the duration of a limited TV-series broadcast as Bass model does. That is, for a social network with the average degree 〈*k*〉, the *average spreading rate* (ASR), denoted as λ, is defined combining with 〈*k*〉 with the mean-field approximation [8, 30]. We also denote *β* as the *average removed rate* (ARR). In addition, based on the Bass model [6], we consider that certain audiences are persuaded by mass media advertising and promotion to start watching with an average probability *α*—*external influence rate* (EIR). Therefore, the differential equations that correspond to the *general imported SIR model* are as follows:

$$\begin{array}{ll} \frac{\partial S\left(t\right)}{\partial t} & =-\alpha S\left(t\right)-\lambda I\left(t\right)S\left(t\right),\\ \frac{\partial I\left(t\right)}{\partial t} & =\alpha S\left(t\right)+\lambda I\left(t\right)S\left(t\right)-\beta I\left(t\right),\\ \frac{\partial R\left(t\right)}{\partial t} & =\beta I\left(t\right). \end{array}$$

where *S*(*t*) + *I*(*t*) + *R*(*t*) = 1. We let *S*(0) = 1, *I*(0) = *R*(0) = 0 be the initial condition.

According to the theory of impulsive differential equations [31], an impulsive-version of the SIR model with a fixed time interval *τ* is as follows:

$$\begin{array}{cc} \frac{\partial S\left(t\right)}{\partial t}=-\alpha S\left(t\right)-\lambda I\left(t\right)S\left(t\right), & t\neq \tau_{k}, \end{array}$$ (1)

$$\begin{array}{cc} \frac{\partial I\left(t\right)}{\partial t}=\alpha S\left(t\right)+\lambda I\left(t\right)S\left(t\right)-\beta I\left(t\right), & t\neq \tau_{k}, \end{array}$$ (2)

$$\begin{array}{cc} \frac{\partial R\left(t\right)}{\partial t}=\beta I\left(t\right), & t\neq \tau_{k}, \end{array}$$ (3)

$$\begin{array}{cc} \Delta I\left(t\right)=\mu I\left(t\right)S\left(t\right), & t=\tau_{k}. \end{array}$$ (4)

where parameter *μ* is the *coefficient of impulse intensity* (CII). Substituting *S*(*t*) + *I*(*t*) + *R*(*t*) = 1 into Eq 1, after rearrangement, we obtain:

$$\begin{array}{c} \frac{\partial S(t)}{\partial t}=\left[\lambda S\left(t\right)-\left(\alpha +\lambda \right)\right]S\left(t\right)+\lambda R\left(t\right)S\left(t\right). \end{array}$$ (5)

Obviously, for a popular TV series, if removed rate *β* ≈ 0, then ⇒*R*(*t*) ≈ 0. Therefore, *I*(*t*) follows an *S-shaped* curve, and *I*(∞) → 1, *S*(∞) → 0. In contrast, for an unpopular TV series, the effect of *β* cannot be ignored. Therefore, the endemic status is *I*(∞) → 0, *S*(∞) → 0, and *R*(∞) → 1. In fact, because no TV series has infinite duration, most *I*(*t*) curves are *J-shaped* during the initial limited broadcast.

The online on-demand streaming statistics are easier to trace and record than the quantity variation of audiences. If we suppose that the newly updated *m* episodes at time *τ*_*k*_ would be viewed by *I*(*t*) agents evenly within the following period *τ*, the *on-demand streaming amount* (ODSA) *V*(*t*) at time *t* has a shape similar to *I*(*t*) and is linear to *I*(*t*):

$$\begin{array}{c} V\left(t\right)=\frac{m}{\tau }I\left(t\right) \end{array}$$ (6)

**Fig 1:**
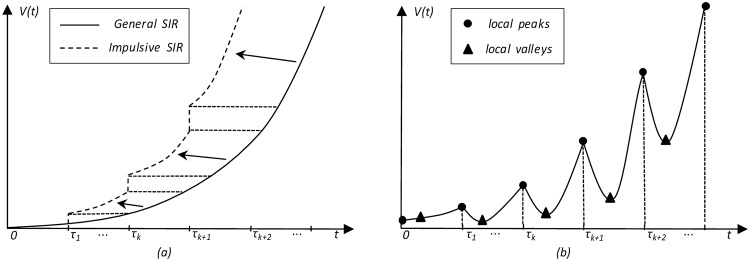
Comparison of the theoretical impulsive SIR diffusion curve and the actual online on-demand streaming curve. (a) The *J-shaped* solid curve indicates the general SIR diffusion process within the initial limited duration, while the piecewise broken curve indicates the step increment effect of impulsive stimulation at every update time *τ*_*k*_. (b) The actual online on-demand streaming curve is a zigzag. Possible explanations are as follows: faithful fans who seek new episodes form the local sharp peaks at every update time *τ*_*k*_ (marked by small round circles); feverish audiences rapidly decrease to form the subsequent valley (marked by small triangles); promotion and buzz cause the slow climb before the next update time *τ*_*k*+1_.

As shown in Fig 1(a), the solid line is the general SIR diffusion curve derived from Eqs 5 and 6, whereas the piecewise broken curve is the general SIR diffusion considering impulsive stimulation at time *τ*_*k*_ of Eq 4. Because the impulsive effect results in a step increment of *I*(*t*), which changes the initial conditions of the next period, the subsequent segment of the impulsive SIR curve will borrow the corresponding segment of the general SIR curve.

However, the actual TV series online ODSA curve is something like Fig 1(b). This zigzagging curve differs from the stepwise impulsive diffusion curve in Fig 1(a). Possible explanations include the following:

1. most audiences, including faithful fans and the newly recruited, are eager for the new episodes and finish watching by every update time, which results in the local sharp peaks (marked by small round circles);
2. a small fraction of the audiences cannot keep pace with new episodes and delays viewing during the next few days, which results in sharp declines after every update time (marked by small triangles);
3. promotion and buzz climb again before the next update time as viewers attempt to guess the plots and attract new viewers to join the audience, which results in the slowly climbing segment after the valley.

### PI-SIR model

**Fig 2:**
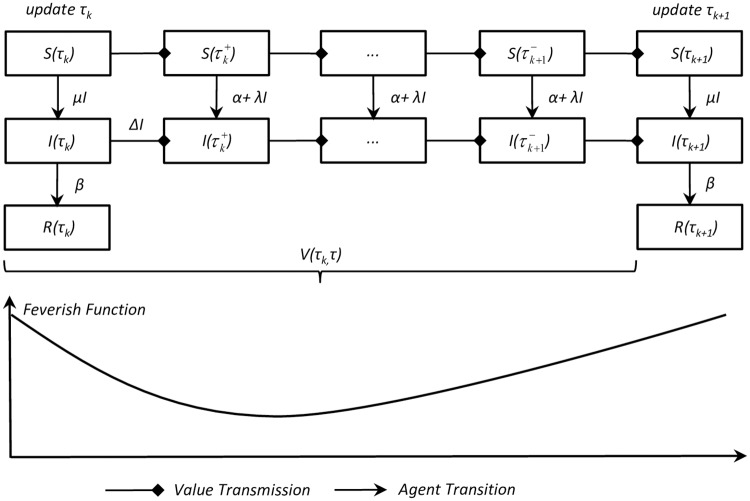
One single-period diffusion process through *τ*_*k*_ to *τ*_*k*+1_. At update time *τ*_*k*_, the impulsive intensity of $\mu S\left(\tau_{k}^{-}\right)I\left(\tau_{k}^{-}\right)$ indicates the newly recruited audience, while removed population $\beta I(\tau_{k}^{-})$ is the audience stopped watching, which is only counted at every update time. During non-update periods, external influence *αS*(*t*) and interpersonal spreading λ*S*(*t*)*I*(*t*) occur in every time unit. A concave feverish function is used to fit the imbalance of the on-demand streaming distribution.

Therefore, we propose a PI-SIR model for online TV series diffusion. Fig 2 illustrates one single-period diffusion process from *τ*_*k*_ to *τ*_*k*+1_.

$$\begin{array}{cc} \frac{\partial S\left(t\right)}{\partial t}=-\frac{\partial I\left(t\right)}{\partial t}=-\alpha S\left(t\right)-\lambda I\left(t\right)S\left(t\right), & t\neq \tau_{k}, \end{array}$$ (7)

$$\begin{array}{cc} \Delta I\left(\tau_{k}\right)=I\left(\tau_{k}\right)-I\left(\tau_{k}^{-}\right)=\mu S\left(\tau_{k}^{-}\right)I\left(\tau_{k}^{-}\right)-\beta I\left(\tau_{k}^{-}\right), & t=\tau_{k}, \end{array}$$ (8)

$$\begin{array}{cc} \Delta R\left(\tau_{k}\right)=R\left(\tau_{k}\right)-R\left(\tau_{k}^{-}\right)=\beta I\left(\tau_{k}^{-}\right), & t=\tau_{k}. \end{array}$$ (9)

Because the audience that stopped watching cannot be tracked during non-update time periods, we count the removed population only at every update time (Eq 9). Therefore, *R*(*τ*_*k*_) is a constant during (*τ*_*k*_, *τ*_*k*+1_), and $S\left(t\right)+I\left(t\right)=\bar{R}\left(\tau_{k}\right)=1-R\left(\tau_{k}\right)$, where $R\left(\tau_{k}\right)=\sum_{i}^{k}\beta I\left(\tau_{i}^{-}\right)$. In addition, we assume that every new audience views the previous episodes, whereas the deposited audience only views the currently updated episodes.

In sum, there are two groups of newly infected agents who view all the *m*(*k*+1) previous episodes in the following period:

1. At update time *τ*_*k*_, $\mu S\left(\tau_{k}^{-}\right)I\left(\tau_{k}^{-}\right)$ susceptible agents transition to infected ones;
2. During *τ*_*k*_ to *τ*_*k*+1_, $\int_{0}^{\tau^{-}}\left[\alpha S(\tau_{k}+i)+\lambda I\left(\tau_{k}+i\right)S\left(\tau_{k}+i\right)\right]di$ susceptible agents transition to infected ones by using a continuous time integral.

Otherwise, at update time *τ*_*k*_, deposited audience $I\left(\tau_{k}^{-}\right)-\beta I\left(\tau_{k}^{-}\right)$ remains, which only views the newly updated *m* episodes during the following period. Therefore, we obtain the *inter-period accumulation* (IPA) of the on-demand streaming amount—*V*(*τ*_*k*_, *τ*) from *τ*_*k*_ to *τ*_*k*+1_:

$$V\left(\tau_{k},\tau \right)=m\left(k+1\right)\left\{\mu S\left(\tau_{k}^{-}\right)I\left(\tau_{k}^{-}\right)+\int_{0}^{\tau^{-}}\left[\alpha S\left(\tau_{k}+i\right)+\lambda I\left(\tau_{k}+i\right)S\left(\tau_{k}+i\right)\right]di\right\}+m\left[I\left(\tau_{k}^{-}\right)-\beta I\left(\tau_{k}^{-}\right)\right].$$ (10)

According to Fig 1(b), we define a concave *feverish function*—*f*(*i*) (Fig 2), which is satisfied by $\int_{0}^{\tau^{-}}f\left(i\right)=1$ to fit the actual zigzag curve.

Finally, the ODSA function *V*(*t*) is:

$$\begin{array}{c} V\left(t\right)=f\left(i\right)V\left(\tau_{k},\tau \right),t=\tau_{k}+i;i=\left[0,\tau \right);k=0,1,\cdots . \end{array}$$ (11)

Different from biological epidemics, the initial audience is always not equal to 0 because of the commercial pre-launch promotion. That is, *I*(0^−^) > 0.

According to the coarse-to-fine strategy, we firstly inspect the inter-period variation of *V*(*τ*_*k*_, *τ*) with respect to *k*. Using variable transformation *i* = *t* − *τ*_*k*_ and substituting Eq 7 into the integral item of Eq 10, yields,

$$\begin{array}{cc} \int_{0}^{\tau^{-}}\left[\alpha S(\tau_{k}+i)+\lambda I\left(\tau_{k}+i\right)S\left(\tau_{k}+i\right)\right]di & =\int_{\tau_{k}}^{\tau_{k+1}^{-}}\frac{\partial I(t)}{\partial t}dt\\ & =I\left(\tau_{k+1}^{-}\right)-I\left(\tau_{k}\right), \end{array}$$ (12)

This result is substituted into Eq 10,

$$\begin{array}{c} V\left(\tau_{k},\tau \right)=m\left(k+1\right)\left[\mu S\left(\tau_{k}^{-}\right)I\left(\tau_{k}^{-}\right)+I\left(\tau_{k+1}^{-}\right)-I\left(\tau_{k}\right)\right]+m\left[I\left(\tau_{k}^{-}\right)-\beta I\left(\tau_{k}^{-}\right)\right] \end{array}$$ (13)

Referring to Eq 8, we have $I\left(\tau_{k}\right)=I\left(\tau_{k}^{-}\right)+\Delta I\left(\tau_{k}\right)=\left(1-\beta \right)I\left(\tau_{k}^{-}\right)+\mu S\left(\tau_{k}^{-}\right)I\left(\tau_{k}^{-}\right)$. This result is Substituted into Eq 13 to yield,

$$\begin{array}{c} V\left(\tau_{k},\tau \right)=m\left[I\left(\tau_{k+1}^{-}\right)-\left(1-\beta \right)I\left(\tau_{k}^{-}\right)\right]k+mI\left(\tau_{k+1}^{-}\right). \end{array}$$ (14)

## Test

### Data collection

In China, Youku.com (www.youku.com), Tudou.com (tv.tudou.com), and iQIYI.com (www.iqiyi.com) are the three leading online video-on-demand suppliers and account for a more than 90% market share. Youku and Tudou merged on Aug. 23*^rd^*, 2012. These two companies publish and update their online streaming statistics data. For the past few years, South Korean TV series have entered the Chinese market and realized a rapid increase in influence. We selected six TV series to test our PI-SIR model: *The Masters Sun* (*Masters*), *The Heirs* (*Heirs*), *My Love From the Stars* (*Love*), *Inspiring Age* (*Age*), *Kaputori*, *Modern Farmer* (*Farmer*). All of the series are broadcast online on Chinese mainland. Therefore, we gather everyday ODSA from the Youku and Tudou websites (http://index.youku.com and http://top.iqiyi.com, respectively). The basic information for these data sets is listed in Table 1.

**Table 1:** Six popular South Korean TV series.

TV Series	*Masters*	*Heirs*	*Love*	*Age*	*Kaputori*	*Farmer*
**launch date** ^×^	2013.08.09	2013.10.11	2013.12.20	2014.1.15	2014.4.11	2014.10.18
**end date** ^×^	2013.10.04	2013.12.13	2014.02.28	2014.4.3	2014.6.21	2014.12.27
**episodes**	17	20	21	24	20	20
**updates** ^+^	9	10	11	12	11	11
**duration** *	60	67	74	81	74	74

^×^ All dates refer to broadcasting date in China’s mainland;

^+^ In China, TV series usually update once every week;

* Duration is days counted from the launch date to three days after the finishing date.

In addition, perform a correlation test of online buzz and on-demand streaming behaviors, we use *Baidu Index* (BI) (http://index.baidu.com/) to collect online buzz longitudinal data. BI is provided by the predominant Chinese search engine company Baidu.com and enables users to search for the search volume and trends of certain hot keywords and phrases.

### Fitting

Based on the datasets, we define an inter-period error function of IPA to estimate the PI-SIR model parameters,

$$\begin{array}{c} err=\sqrt{\frac{\sum_{k=1}^{U}{\left[\frac{V\left(\tau_{k},\tau \right)-V^{{}^{\prime }}\left(\tau_{k},\tau \right)}{\sum_{t=1}^{D}V\left(t\right)}\right]}^{2}}{U}}, \end{array}$$ (15)

where *D* is the number of duration, *U* is the number of updates and equal to the floor integer value of $\frac{D}{\tau }$, *V*(*τ*_*k*_, *τ*) is the actual value and *V*′(*τ*_*k*_, *τ*) is the fitting value.

Based on a report on China’s online video streaming market, the total number of potential audience members was about *N* = 4.0*e* + 8 in 2013. A two-step fitting scheme is used to seek out the estimated parameters. In the first step, we harvest the estimated values of $\hat{\alpha }$, $\hat{\lambda }$, $\hat{\beta }$, and $\hat{\mu }$ by,

$$\begin{array}{c} \left[\hat{\alpha },\hat{\lambda },\hat{\beta },\hat{\mu }\right]=\arg\min_{V(\tau_{k},\tau )}err. \end{array}$$ (16)

Based on the three samples, the remaining constants are established as *τ* = 7, *m* = 2. We believe the initial audience to be $I^{-}\left(0\right)=\frac{V(0)}{m}$. The estimated parameters of $\hat{\alpha }$, $\hat{\lambda }$, $\hat{\beta }$, and $\hat{\mu }$ are listed in Table 2. Then, before the second step, we use *cosine similarity* to investigate whether the inner-period quasi-parabolic curve segments have similar characteristics,

$$\begin{array}{c} sim\left(j,k\right)=\frac{\left\langle v_{j},v_{k}\right\rangle }{∥v_{j}∥\cdot ∥v_{k}∥}=\frac{\sum_{i=1}^{\tau }V\left(\tau_{j},i\right)V\left(\tau_{k},i\right)}{\sqrt{\sum_{i=1}^{\tau }V^{2}\left(\tau_{j},i\right)}\cdot \sqrt{\sum_{i=1}^{\tau }V^{2}\left(\tau_{k},i\right)}}, \end{array}$$ (17)

where ***v*** *_k_* = [*V* (*τ_k_*, 1), …, *V* (*τ_k_*, *τ*)] refers to the vector of the *k*^*th*^ period. The *similarity mean* and *coefficient of variance* are,

$$\begin{array}{cc} \bar{sim} & =\frac{2\sum_{j=1}^{U-1}\sum_{k=j+1}^{U}sim\left(j,k\right)}{U\left(U-1\right)}, \end{array}$$ (18)

$$\begin{array}{cc} V_{s} & =\sqrt{\frac{2\sum_{j=1}^{U-1}\sum_{k=j+1}^{U}{\left[\bar{sim}-sim\left(j,k\right)\right]}^{2}}{U\left(U-1\right)\cdot {\bar{sim}}^{2}}}. \end{array}$$ (19)

**Table 2:** Estimated parameters of the six datasets.

TV Series	*Masters*	*Heirs*	*Love*	*Age*	*Kaputori*	*Farmer*
**EIR($\hat{\alpha }$)**	0.00020	0.00020	0.00007	0.00001	0.00001	0.00001
**ARR($\hat{\beta }$)**	0.1000	0.1000	0.0988	0.8002	0.2802	0.9902
**ASR($\hat{\lambda }$)**	0.2970	0.0439	0.0497	0.0201	0.0401	0.2801
**CII($\hat{\mu }$)**	0.0020	0.0020	0.2360	0.0160	0.0020	0.0220
$\hat{f}\left(1\right)$	0.2280	0.2597	0.2225	0.0719	0.1099	0.1251
$\hat{f}\left(2\right)$	0.1579	0.1615	0.1409	0.1894	0.1564	0.1468
$\hat{f}\left(3\right)$	0.1266	0.1194	0.1217	0.2320	0.2167	0.1617
$\hat{f}\left(4\right)$	0.1010	0.0826	0.0918	0.1966	0.1697	0.1428
$\hat{f}\left(5\right)$	0.0914	0.0753	0.0917	0.1596	0.1346	0.2092
$\hat{f}\left(6\right)$	0.0884	0.0736	0.0925	0.1281	0.1081	0.0965
$\hat{f}\left(7\right)$	0.2066	0.2279	0.2389	0.0220	0.1050	0.1180

**Fig 3:**
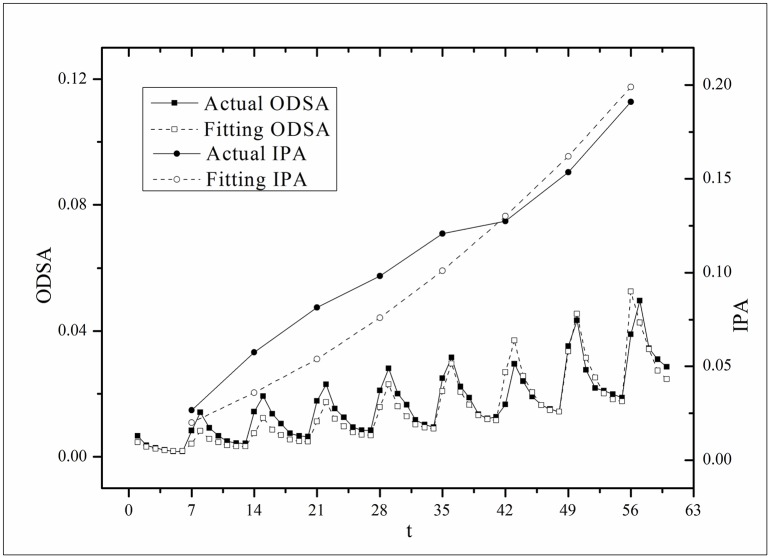
Two-step fitting results for the *Masters* dataset.

**Fig 4:**
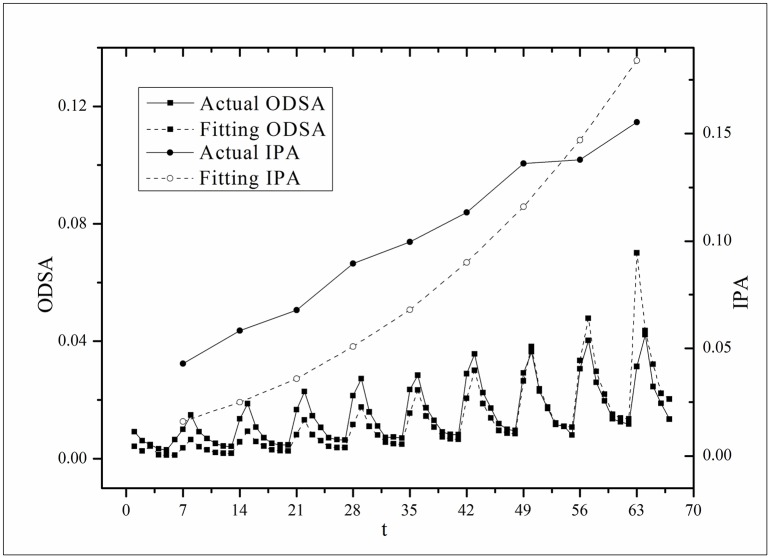
Two-step fitting results for the *Heirs* dataset.

**Fig 5:**
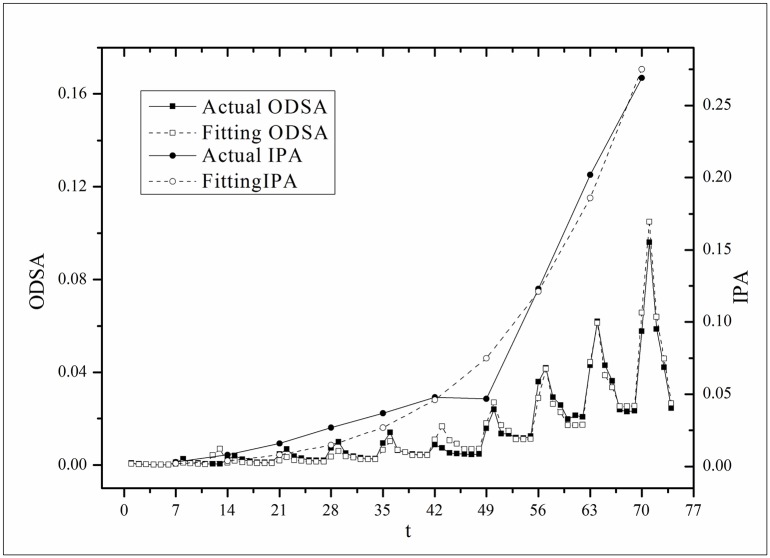
Two-step fitting results for the *Love* dataset.

**Fig 6:**
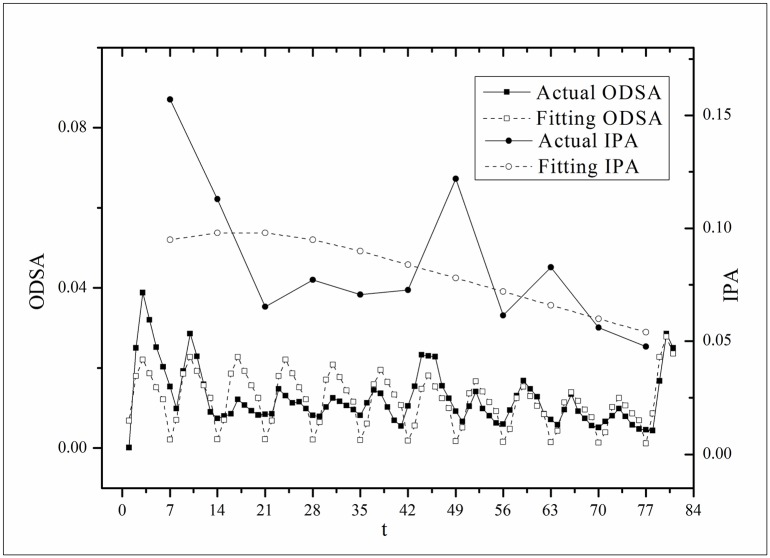
Two-step fitting results for the *Age* dataset.

**Fig 7:**
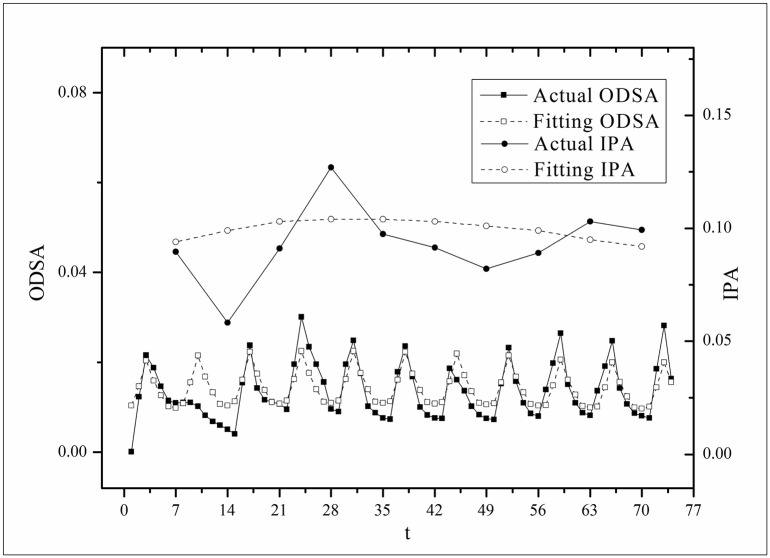
Two-step fitting results for the *Kaputori* dataset.

**Fig 8:**
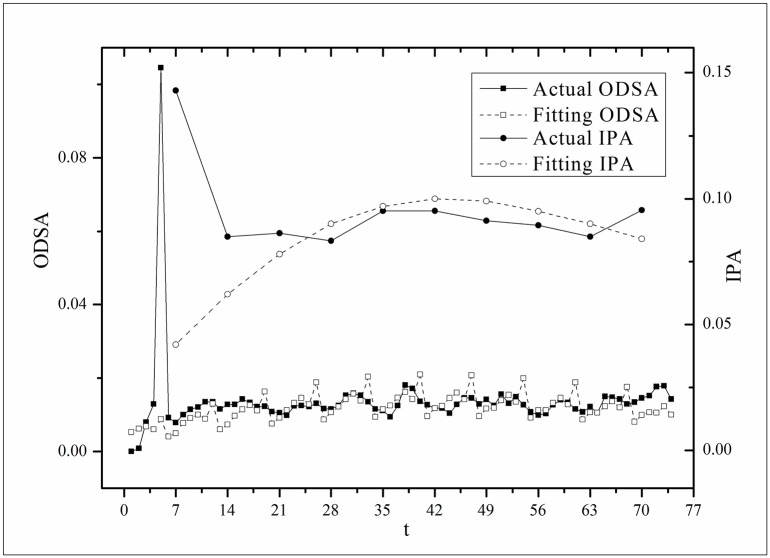
Two-step fitting results for the *Farmer* dataset.

As listed in Table 3, the six $\bar{sim}\text{s}$ are almost larger than 0.9, and the *V*_*s*_s are very close to 0 (except *Farmer*’s), which implies that the ODSA of every period might exhibit the same characteristics from a perspective of collective behavior. Therefore, we can only use one set of estimated parameters of $\hat{f}\left(\cdot \right)\text{s}$ to fit the feverish function in the second step as follows:

$$\begin{array}{c} \hat{f}\left(i\right)=\frac{1}{U}\cdot \sum_{k=1}^{U}\frac{V(\tau_{k}+i)}{V(\tau_{k},\tau )},\;\sum_{i=1}^{\tau }\hat{f}\left(i\right)=1;\sum_{i=1}^{\tau }V^{{}^{\prime }}\left(\tau_{k}+i\right)=V^{{}^{\prime }}\left(\tau_{k},\tau \right);i=1,...,\tau . \end{array}$$ (20)

where *V*(*τ*_*k*_ + *i*) and *V*(*τ*_*k*_, *τ*) are the actual values and *V*′(*τ*_*k*_ + *i*) and *V*′(*τ*_*k*_, *τ*) are the fitting values. The results for $\hat{f}\left(\cdot \right)\text{s}$ are listed in Table 2. Figs 3–8 show the two-step fitting results for the six datasets.

**Table 3:** Similarity and correlation coefficients of the six datasets.

TV Series	*Masters*	*Heirs*	*Love*	*Age*	*Kaputori*	*Farmer*
$\bar{sim}$	0.980	0.992	0.964	0.973	0.966	0.896
***V*_*s*_**	0.022	0.010	0.028	0.023	0.031	0.211
***Corr***	0.870	0.954	0.938	0.642	0.746	0.066

**Fig 9:**
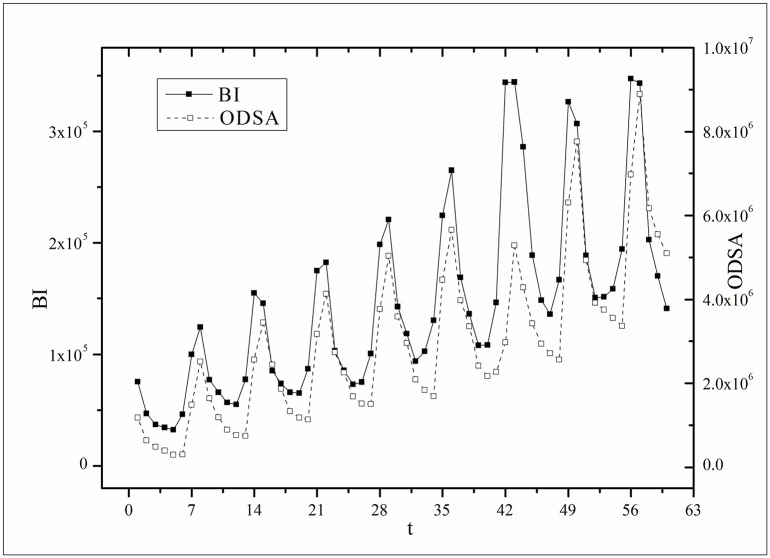
Correlation analysis of the *Masters* dataset.

**Fig 10:**
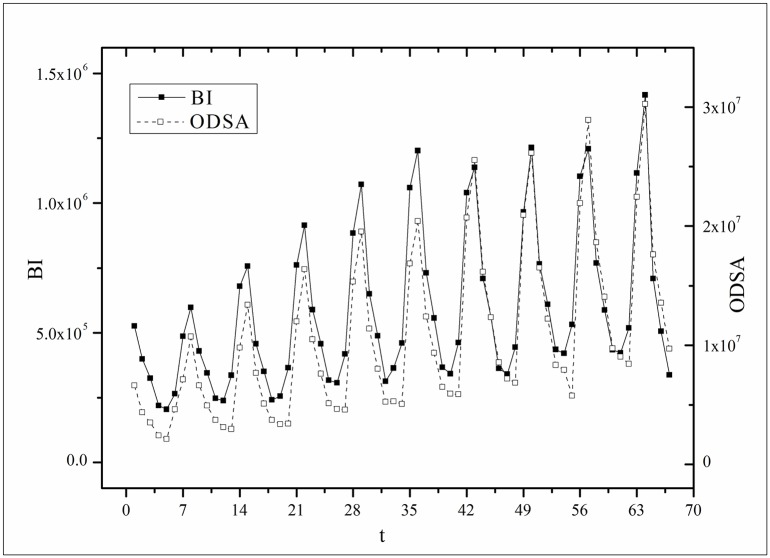
Correlation analysis of the *Heirs* dataset.

**Fig 11:**
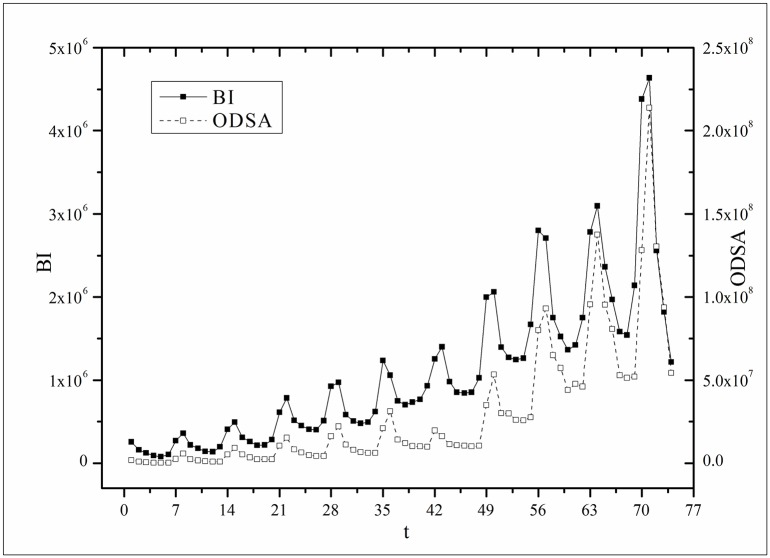
Correlation analysis of the *Love* dataset.

**Fig 12:**
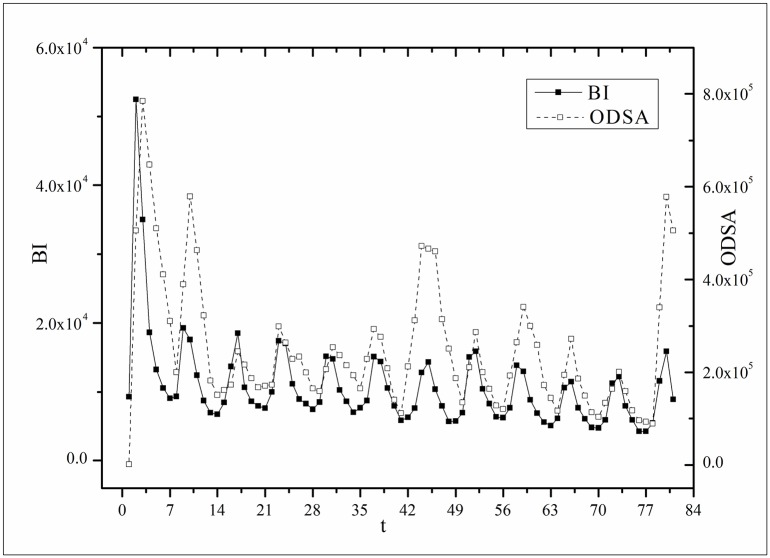
Correlation analysis of the *Age* dataset.

**Fig 13:**
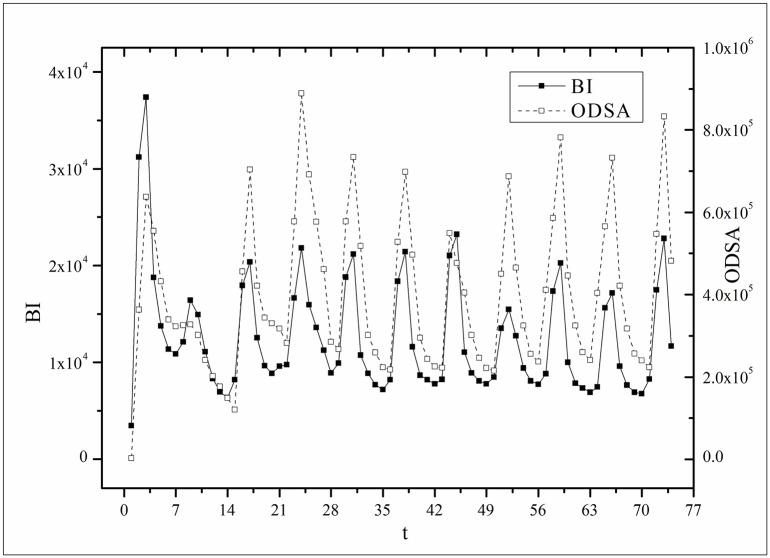
Correlation analysis of the *Kaputori* dataset.

**Fig 14:**
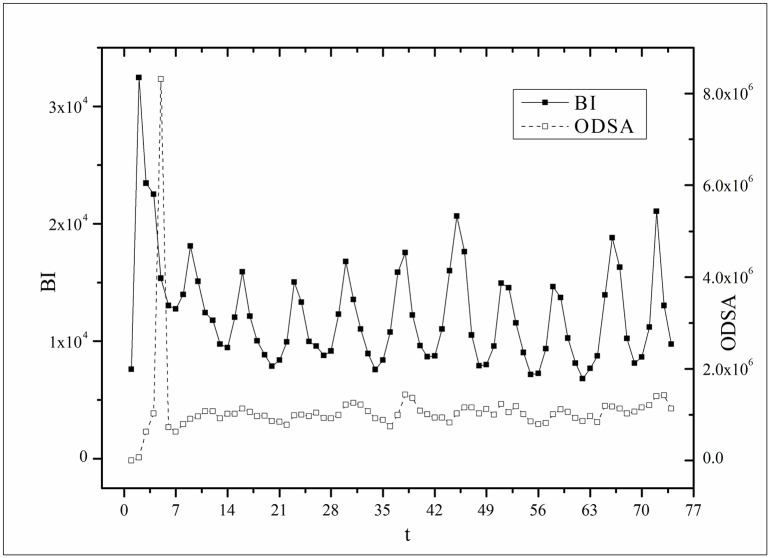
Correlation analysis of the *Farmer* dataset.

Finally, the *Pearson correlation coefficient* is used to investigate the correlation between word-of-mouth and on-demand streaming,

$$\begin{array}{ll} Corr\left(V\left(t\right),B\left(t\right)\right) & =\frac{\langle V\left(t\right)-\bar{V},B\left(t\right)-\bar{B}\rangle }{\left\|V\left(t\right)-\bar{V}\|\cdot \|B\left(t\right)-\bar{B}\right\|}\\ & =\frac{\sum_{t=1}^{D}\left[V\left(t\right)-\bar{V}\right]\left[B\left(t\right)-\bar{B}\right]}{\sqrt{\sum_{t=1}^{D}{\left[V\left(t\right)-\bar{V}\right]}^{2}}\cdot \sqrt{\sum_{t=1}^{D}{\left[B\left(t\right)-\bar{B}\right]}^{2}}}, \end{array}$$ (21)

where *B*(*t*) is the BI temporal series that corresponds to the broadcasting periods and $\bar{V}$ and $\bar{B}$ are the mean values of *V*(*t*) and *B*(*t*), respectively. The results of *Corr* are listed in Table 3. Figs 9–14 show the fluctuation correlation of the six datasets.

### Findings and Discussion

Our comprehensive analysis of the six datasets yields five findings as follows.

**Fig 15:**
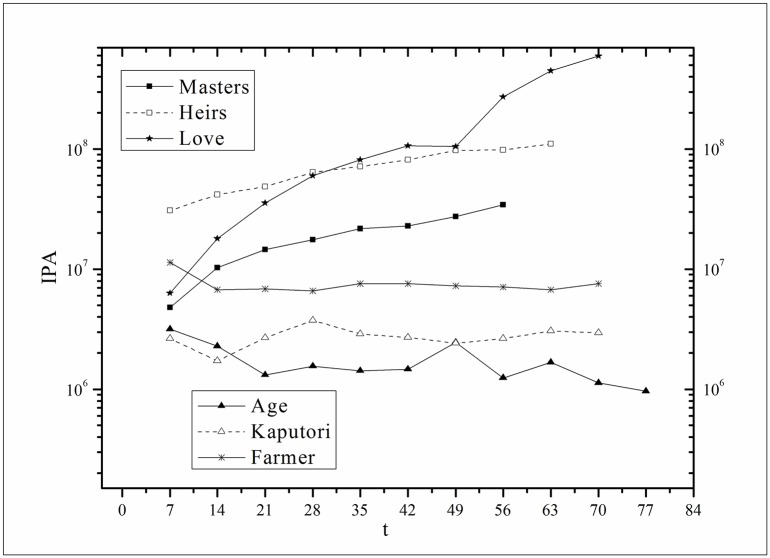
Comparison of IPA of the six datasets.

**Fig 16:**
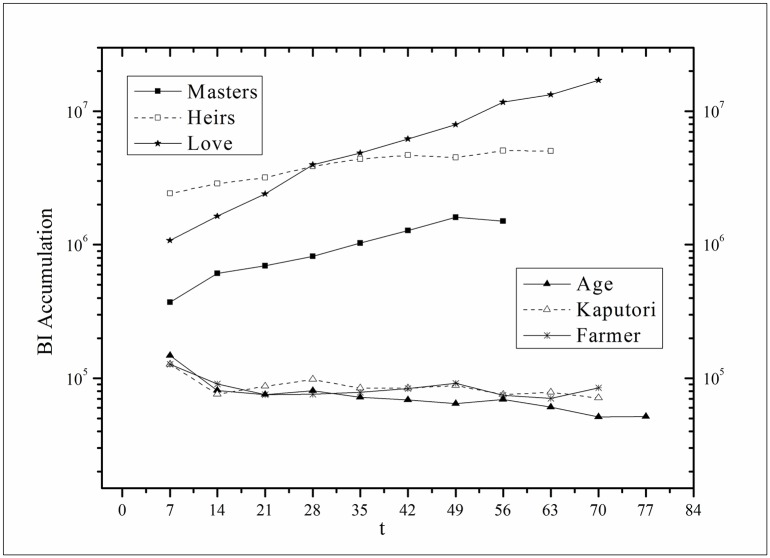
Comparison of BI accumulation of the six datasets.

**Fig 17:**
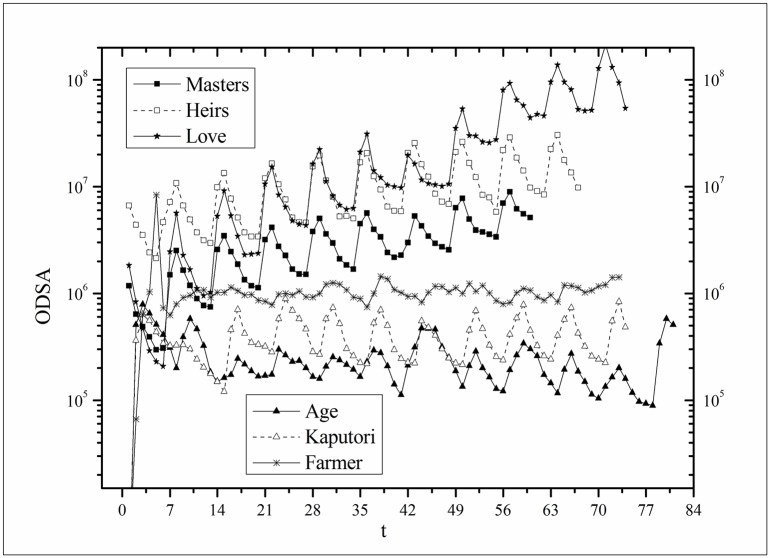
Comparison of ODSA of the six datasets.

**Fig 18:**
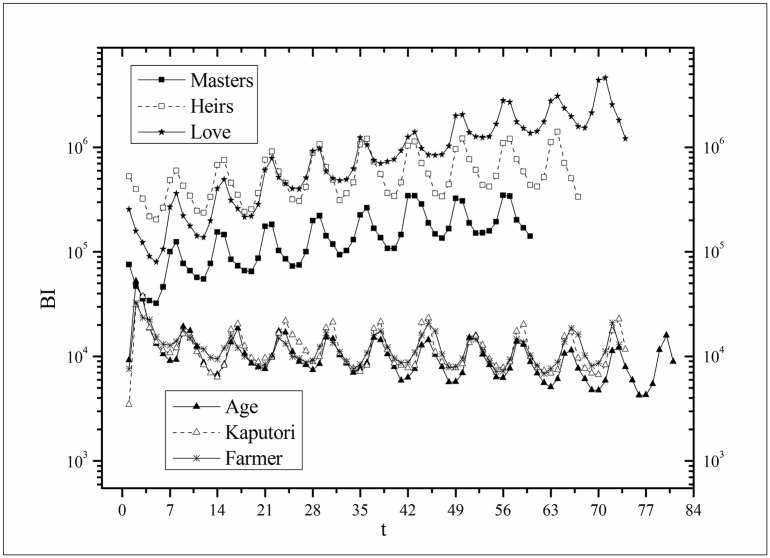
Comparison of BI of the six datasets.

1. Audiences have similar viewing habits. Comparing $\hat{f}\left(1\right)$ – $\hat{f}\left(7\right)$ in Table 2, we find that the feverish distributions within an update period of six different types of TV series are periodically fluctuated, which means that the audience attention imbalance affected by externally impulsive stimulation.
2. The on-demand streaming fluctuation is highly correlated with the online buzz fluctuation. According to an empirical criterion of PCC, the first 5 *Corr*s in Table 3 are larger than 0.5, which indicates high correlation. Visual confirmation in Figs 9–13 supports this finding. The explanation might be that today’s audience is accustomed to search for information, to post messages, to discuss plots, and to exchange ideas in online social communities when they are watching TV series. However, the *Corr* of *Farmer* is close to 0, which indicates no correlation. By investigating the two curves in Fig 14, we find that the mismatched and high weighted initial peaks of BI and OSDA curves might bring *Corr* down, although ODSA waves after BI slightly.
3. Impulsive intensity plays a crucial role. Comparing the EIRs, ARRs, ASRs, and CIIs of *Love* and *Heirs* in Table 2, we find that the CII of *Love* is approximately 118 times larger than that of *Heirs*, while the EIR, ARR, and ASR of the two series are similar. Although the initial ODSA and initial BI of *Love* listed in Table 4 are less than those of *Heirs*, the ODSA and BI of *Love* climb much faster than those of *Heirs*. The ODSA and BI of *Love* start to exceed *Heirs* from the 5*^th^* week, which can be deduced from the IPA curves shown in Fig 15, the BI accumulation curves shown in Fig 16, the ODSA fluctuation curves shown in Fig 17, and the BI fluctuation curves shown in Fig 18. Finally, according to Table 4, the total ODSA of *Love* is approximately 3 times larger than that of *Heirs*, and the BI of *Love* is approximately 2 times larger than that of *Heirs*.
4. Audience activity degree indicates the trend of streaming rate. We can divide the six series into two groups. The first group includes *Masters*, *Heirs*, and *Love*. The second group includes *Age*, *Kaputori*, and *Farmer*. The BI accumulation values of the first group are tens or hundreds of times larger than those of the second group shown in Fig 16. This indicates audience activity degree for the first group is greatly larger than that of the second group. Therefore, comparing the EIR, ARRs, ASRs, and CIIs in Table 2, we find the removed rates of the second group are several times larger than those of the first group. High ARRs depress the rising of ODSA curves, even drive the trend down (see the IPA curve in Fig 15).
5. The initial value is also important. Comparing the EIRs, ARRs, ASRs, and CIIs of *Heirs* and *Masters* in Table 2, we find that the ASR of *Masters* is approximately 6.7 times larger than that of *Heirs*, while the other three parameters are equal. This outcome explains why two ODSA curves exhibit a similar shape (Fig 17). However, the ODSA curve of *Masters* climbs slightly more than that of *Heirs* at the tail because of the larger ASR value. As listed in Table 4, the total ODSA of *Heirs* is approximately 4 times larger than that of *Masters* and the total BI of *Heirs* is approximately 4.5 times larger than that of *Masters*. A significant reason for this gap is the initial value. Also with respect to Table 4, the initial ODSA of *Heirs* is approximately 5.6 times larger than that of *Masters*, while the initial BI of *Heirs* is approximately 6.9 times larger than that of *Masters*. In addition, the initial values of *Age*, *Kaputori*, and *Farmer* are greatly less than those of the other three series. Therefore, their performance of total ODSA and BI are also worse.

**Table 4:** Comparison of other basic parameters of the six datasets.

TV Series	*Masters*	*Heirs*	*Love*	*Age*	*Kaputori*	*Farmer*
**Total ODSA**	1.79e+8	7.17e+8	2.22e+9	2.02e+7	2.96e+7	7.96e+7
**Total BI**	8.76e+6	3.91e+7	8.05e+7	8.65e+5	9.29e+5	8.98e+5
**Initial ODSA**	1.18e+6	6.63e+6	1.82e+6	1.49e+3	2.68e+3	3.13e+3
**Initial BI**	7.56e+4	5.25e+5	2.55e+5	9.26e+3	3.47e+3	7.61e+3

## Conclusion

According to our PI-SIR model, the online on-demand streaming amount of TV series fluctuates with respect to the periodic impulsive stimulation and climbs as word-of-mouth diffuses. Because the audience can optionally review previous episodes whenever and wherever possible in the context of online streaming, a feature that traditional TV broadcasts cannot provide, the word-of-mouth effect, accumulates to amplify the audience rating. Our analysis results for six different types of South Korean TV series reveal that impulsive intensity on update days and pre-launch promotion have stronger impacts on the total on-demand streaming amount than other parameters. The implication of these results for management is that it is worthwhile to invest in promotion near update days to stimulate audience attention and interpersonal diffusion. In addition, strong pre-launch promotion seems to be a good marketing tool to improve overall performance. Our research also reveals that it is not advisable for online video providers to promote several popular TV series on the same update day because of the imbalanced distribution of audience intention. The technical implication of our research is that inter-period accumulation is a feasible forecasting tool to predict the future trend of the on-demand streaming amount. In addition, the buzz in public social communities is a highly correlated analysis tool to evaluate the advertising value of TV series.

## Limitations

Our model seems not good at fitting emergency situations because differential equations try to smooth nonlinear trends. Today, as big data can be found everywhere, online video on-demand streaming providers would like to develop technologies to trace user habits using cookies or other behavioral footprints. In this research, we could obtain more detailed information except through the open records of on-demand streaming amounts. In future research, big data and more intelligent technologies can be expected to fill this gap.

## Supporting Information

S1 File *The Masters Sun* Dataset. Daily record of buzz volume in *Baidu Index* and total online streaming quantity on the Youku-Tudou and iQiYi platforms for the South Korean TV series *The Masters Sun* during broadcasting.
(TXT)

S2 File *The Heirs* Dataset. Daily record of buzz volume in *Baidu Index* and total online streaming quantity on the Youku-Tudou and iQiYi platforms for the South Korean TV series *The Heirs* during broadcasting.
(TXT)

S3 File *My Love From the Stars* Dataset. Daily record of buzz volume in *Baidu Index* and total online streaming quantity on the Youku-Tudou and iQiYi platforms for the South Korean TV series *My Love From the Stars* during broadcasting.
(TXT)

S4 File *Inspiring Age* Dataset. Daily record of buzz volume in *Baidu Index* and total online streaming quantity on the Youku-Tudou and iQiYi platforms for the South Korean TV series *Inspiring Age* during broadcasting.
(TXT)

S5 File *Kaputori* Dataset. Daily record of buzz volume in *Baidu Index* and total online streaming quantity on the Youku-Tudou and iQiYi platforms for the South Korean TV series *Kaputori* during broadcasting.
(TXT)

S6 File *Modern Farmer* Dataset. Daily record of buzz volume in *Baidu Index* and total online streaming quantity on the Youku-Tudou and iQiYi platforms for the South Korean TV series *Modern Farmer* during broadcasting.
(TXT)
